# Physiological dissociation between ventilatory ratio and ventilatory efficiency in patients with ARDS

**DOI:** 10.1186/s40635-026-00887-4

**Published:** 2026-03-19

**Authors:** Martín H. Benites, Fernando Suarez‑Sipmann, Arnoldo Santos, Jaime Retamal

**Affiliations:** 1 Centro de Pacientes Críticos, Clínica Las Condes, Santiago, Chile; 2 Departamento de Medicina Intensiva, Facultad de Medicina, Pontificia Universidad Católica de Chile, Santiago, Chile; 3 Facultad de Medicina, Escuela de Medicina, Universidad Finis Terrae, Santiago, Chile; 4 Programa de Doctorado en Ciencias Médicas, Escuela de Medicina, Pontificia Universidad Católica de Chile, Santiago, Chile; 5 CIBER de Enfermedades Respiratorias, Instituto de Salud Carlos III, Madrid, Spain; 6 Intensive Care Medicine Department, Hospital Universitario de La Princesa, Madrid, Spain; 7 Department of Surgical Sciences, Uppsala University, Uppsala, Sweden; 8 Intensive Care Medicine Department, Hospital Universitario Fundación Jiménez Díaz, Instituto de Investigación Sanitaria Fundación Jiménez Díaz, Madrid, Spain

**Keywords:** Acute respiratory distress syndrome, Alveolar ventilation, Bohr dead space, Ventilatory efficiency, Ventilatory ratio, Volumetric capnography

## Abstract

**Background:**

The ventilatory ratio (VR) is frequently used as a surrogate marker of ventilatory efficiency in patients with ARDS. However, its ability to reflect changes in alveolar ventilation (V̇Talv/VT) when respiratory mechanics are modified remains unknown. This study aimed to evaluate the relationship between VR and V̇Talv/VT during sequential changes in respiratory mechanics=, tidal volume (VT), and minute ventilation (V̇E) in patients with ARDS.

**Methods:**

This was a secondary analysis of a quasi-experimental, repeated-measures study conducted in a single-center adult ICU. Twenty-two patients with ARDS were evaluated across three sequential 60 min controlled periods, during which trunk inclination was adjusted to induce changes in VT. At the end of each period, VR was calculated, and V̇Talv/VT was measured using volumetric capnography. A total of 66 paired measurements were analyzed in this study.

**Results:**

By design, VT increased from Time 1 to Time 2 by + 62 mL and decreased from Time 2 to Time 3 by − 68 mL. These changes in VT were associated with the following: VR was not significantly different between Time 1 and Time 2 [− 0.23 (95% CI: − 0.44 to − 0.02; p = 0.071)] or between Time 2 and Time 3 [+ 0.17 (95% CI: − 0.04 to + 0.38; p = 0.086)].

The alveolar ventilation ratio (V̇Talv/VT) increased significantly from Time 1 to Time 2 by + 0.080 (95% CI: + 0.039 to + 0.121; p < 0.001), and decreased from Time 2 to Time 3 by − 0.060 (95% CI: − 0.101 to − 0.019; p < 0.001). Association between VR and V̇Talv/VT: no significant relationship was found (β =  − 0.056, marginal R^2^ = 0.052, conditional R^2^ = 0.205, p = 0.111).

**Conclusions:**

In this cohort of patients with ARDS, VR did not correlate with V̇Talv/VT following controlled modifications of respiratory mechanics. These findings suggest that VR may not reliably represent ventilatory efficiency under changing ventilatory conditions, and its use as a surrogate variable should be approached with caution.

## Background

The ventilatory ratio (VR) was introduced into clinical practice as a surrogate marker of ventilatory efficiency in patients with acute respiratory distress syndrome (ARDS) [1]. Since then, the vast majority of studies have shown that increases in VR are consistently associated with adverse clinical outcomes, including increased mortality [2–4]. However, it remains unclear whether VR accurately reflects the physiological mechanisms underlying ventilatory efficiency in ARDS.

Conceptually, ventilatory efficiency refers to the ability of the lungs to eliminate carbon dioxide (CO₂) during each respiratory cycle. It is commonly expressed as the proportion of each breath that participates in the alveolar ventilation (V̇Talv/VT) [1]. To compute alveolar ventilation, it is necessary to determine the relationship between the exhaled volume of CO_2_ per breath (V̇TCO_2,br_) and mean alveolar CO₂ pressure (PACO_2_) [5]. Instead, the VR is computed using mean arterial CO₂ pressure (PaCO_2_) measurements based on the ideal alveolar gas equation [1]. However, in mechanically ventilated patients with ARDS, alveolar gas is far from ideal, as PaCO_2_ reflects the effects of ventilation–perfusion (V/Q) mismatch, including venous admixture and increased dead space [6, 7]. Likewise, VR has been validated as an index of ventilatory efficiency using the Enghoff dead-space fraction as a reference standard [2, 8], which, like VR, replaces PACO_2_ with PaCO_2_ [7] and therefore presents the same physiological limitations.

Furthermore, VR embeds fixed “normal” constants in its denominator (e.g., a standardized minute ventilation (V̇E) per predicted body weight derived from anesthetized, non-ARDS populations), which may not be appropriate in ARDS and can bias the interpretation across phenotypes and ventilatory settings [9]. This undermines the assumption that VR reliably represents ventilatory efficiency under changing ventilatory conditions. Consistent with this, the VR has not been physiologically validated against capnography-based measures of ventilatory efficiency (e.g., V̇Talv/VT) or established indices of ventilatory inefficiency (e.g., Bohr dead space fraction) during changes in respiratory mechanics.

We hypothesized that VR would not consistently reflect dynamic changes in ventilatory efficiency when tidal volume (VT) and V̇E are modified. Accordingly, the objective of this study was to assess how sequential changes in respiratory mechanics, along with the resulting modifications in VT and V̇E, affect VR and V̇Talv/VT in ARDS patients.

## Methods

### Study design

We conducted a secondary analysis of a previously published quasi-experimental study with a repeated measures design. The analytic sample comprised 22 adults (≥ 18 years) with ARDS who received invasive mechanical ventilation for < 7 days under deep sedation and/or neuromuscular blockade [10]. The original study was approved by the ethics committee of Clínica las Condes (protocol number: E01202) and registered as NCT05281536 in ClinicalTrials.gov.

### Study methods

In the study protocol, trunk inclination was sequentially adjusted from 45° (Time 1, baseline) to 10° (Time 2, VT increase) and then returned to 45° (Time 3, recovery), resulting in consistent changes in VT and V̇E without changes in the driving pressure, PEEP, or sedation level. Each position was maintained for 60 min, during which the flow and exhaled CO_2_ signals were recorded using volumetric capnography, and V̇E was calculated. The final 45° period (Time 3) served as a control to confirm the reversibility of the changes observed at Time 2.

### Volumetric capnography

We used the Fluxmed monitor (MBMed) in this study, featuring a mainstream infrared sensor (Capnostat 5^®^; Respironics, OH, USA) capable of measuring expired CO_2_ (range 0–150 mmHg), with an accuracy of ± 2 mmHg and a response time of less than 60 ms. This sensor was integrated with a Fluxmed monitor via an MBMed CO_2_ module. The device incorporates MATLAB-programmed software (MathWorks, Natick, MA, USA) to analyze ventilation in volumetric capnograms in real-time, utilizing a mathematical algorithm (Levenberg–Marquardt) that matches VT with exhaled CO_2_. Consequently, it was feasible to derive data on dead space and alveolar ventilation. Data were recorded using Fluxview^®^, a customized analysis software [10]. From the synchronized flow and CO_2_ signals, we calculated the following:

### Area under the capnogram curv.

- **CO**_**2**_** eliminated per breath (V**_**T**_**CO**_**2**_**,**_**br**_**):** Expired CO₂ volume represents the area under the capnogram curve and quantifies the amount of carbon dioxide eliminated per breath (mL).
- **Minute CO**_**2**_** output (V̇CO**_**2**_**):** V_T_CO_2_,_br_ × respiratory rate (RR).
- **Fraction of expired CO₂ (F**_**E**_**CO**_**2**_**):** V_T_CO_2_,_br_/expired VT; Represents the mean amount of CO₂ diluted in each expired tidal volume.
- **Mean expired CO**_**2**_** pressure (P**_**E**_**CO**_**2**_**):** F_E_CO_2_ × (barometric pressure − water vapor pressure) [10, 11].

### Dead space and alveolar ventilation

- **Bohr dead space fraction (VD**_**Bohr**_**/VT):** (PACO_2_−P_E_CO_2_) / PACO_2_, where PACO_2_ is the mean alveolar CO_2_ tension at the midpoint of phase III of the capnogram. It represents the relationship between the physiological dead space (airway dead space (VDaw) + alveolar dead space (VDalv)) and expired VT. This index quantifies the proportion of expired tidal volume that does not participate in effective gas exchange, reflecting overall ventilatory inefficiency.
- **Physiological dead space or Bohr dead space (mL)** was obtained by multiplying the VD_Bohr_/VT fraction by the expired VT.
- **Airway dead space (VDaw) (mL)**: It was computed in the mind point phase II of each capnogram
- **Alveolar dead space (VDalv) (mL):** This is derived by subtracting VDaw from physiological dead space
- **Alveolar ventilation fraction (V̇Talv/VT):** alveolar VT/expired VT. V̇Talv = (VT−VDaw)
- **Alveolar minute ventilation (V̇A) (ml/min)** was computed as V̇Talv × RR and indexed to the PBW (predicted body weight).
- **Effective alveolar ventilation:** As alveolar ventilation includes a proportion of volume that does not contain CO_2_, corresponding to the alveolar dead space, the following formula was applied: effective V̇Talv = (V̇Talv–VD_alv_) [10]. From this variable arises **V̇Talv/VTeffective** [11, 12]. This index quantifies the proportion of expired tidal volume that does participate in effective gas exchange, reflecting overall ventilatory efficiency.

### Global gas-exchange metrics

○ **Enghoff index gas exchange (VD**_**Enghoff**_**/VT): (**PaCO_2_–PECO_2_)/PaCO_2_; incorporates the effects of V̇/Q̇ mismatch, including all low and high V/Q units and their extremes, shunt, and dead space.
○ **Phase III slope (SIII):** steepness of phase III of the capnogram; sensitivity to V/Q̇ heterogeneity. We also report the normalized slope (SnIII = SIII/ F_E_CO_2_) to allow comparison across breaths with different CO_2_ excretion rates, which is expected when the VT changes [10, 11].

All signals were exported and analyzed offline; for each condition, the mean of the last 20 breaths was calculated.

### Ventilatory ratio (VR)

VR was calculated as (V̇E × PaCO_2_)/(PBW kg × 100 mL·kg⁻^1^·min⁻^1^ × 37.5 mmHg), where V̇E is minute ventilation and PBW is predicted body weight [2].

### Outcome

The primary outcome of this study was to assess the association between the ventilatory ratio (VR) and alveolar ventilation fraction (V̇Talv/VTeffective).

### Statistical analysis

Continuous variables are expressed as the mean and standard deviation (± SD) and were analyzed using ANOVA for repeated measures. The Bonferroni correction post hoc test was used to compare the different study steps. The F statistic and its corresponding p-value were reported to assess the overall effect of time. When ANOVA indicated statistical significance, a post hoc analysis was performed using pairwise contrasts based on estimated marginal means (EMMeans) to identify specific differences across time points (comparisons T1 vs. T2, T1 vs. T3, and T2 vs. T3). For each contrast, the adjusted p-value was reported. Marginal and conditional R^2^ values were calculated to quantify the proportion of variance explained by the fixed effects and full mixed-effects models. The strength of the associations was evaluated using regression coefficients, and statistical significance was defined as p < 0.05. Additionally, concordance was assessed using four-quadrant plots to evaluate directional agreement in paired changes in variables over time. Statistical analyses were conducted using RStudio version 4.5.1 (Integrated Development Environment; Boston, MA, USA).

## Results

A total of 66 sets of measurements (22 patients × 3 phases) of VR and volumetric capnography–derived variables were analyzed. Table 1 summarizes the results of respiratory mechanics, arterial blood gases, and volumetric capnography at the study time points.

**Table 1:** Results of respiratory mechanics, arterial blood gases, and volumetric capnography

	T 1 (Mean ± SD)	T 2 (Mean ± SD)	T 3 (Mean ± SD)	F (Time)	*p*-value	T1–T2 (*p*)	T1–T3 (*p*)	T2–T3 (*p*)
VT (mL)	371 ± 76.2	433 ± 84.9	365 ± 78.1	105.7	< 0.001	< 0.001	0.763	< 0.001
RR (breaths·min⁻^1^)	21 ± 2	21 ± 2	21 ± 2.9	0.956	0.393	0.976	0.703	1.000
V̇E (L · min⁻^1^)	8 ± 1.1	9.3 ± 1.2	7.9 ± 1.1	102.8	< 0.001	< 0.001	0.742	< 0.001
V̇E (mL·kg⁻^1^·min⁻^1^)	129 ± 26	149 ± 30	127 ± 26	91.19	< 0.001	< 0.001	0.851	< 0.001
C_RS_ (mL cmH_2_0)	35.0 ± 10.5	41.6 ± 12.4	34.4 ± 10.31	79.84	< 0.001	< 0.001	1.000	< 0.001
PEEP (cmH_2_0)	10.5 ± 1.4	10.5 ± 1.4	10.5 ± 1.47	1.711	0.193	0.911	1.000	1.000
PaO_2_/F_I_O_2_ (mmHg)	189 ± 33	196 ± 34	191 ± 29	2.78	0.0733	0.0778	1.000	0.370
PaCO_2_ (mmHg)	43.3 ± 5.1	36.0 ± 4.3	42.7 ± 5.34	89.65	< 0.001	< 0.001	0.999	< 0.001
VT_alv_ (mL)	227 ± 65.2	303 ± 84	246 ± 76	92.51	< 0.001	< 0.001	0.0063	< 0.001
VT_alv_ (mL·min^−1^)	4642 ± 918	6201 ± 1196	5023 ± 1086	101.3	< 0.001	< 0.001	0.0053	< 0.001
VT_alv_ (mL·kg⁻^1^·min⁻^1^)	73 ± 16	98 ± 21	79 ± 18	101.4	< 2e-16	< 0.0001	0.0063	< 0.0001
PACO_2_ (mmHg)	37 ± 4.3	31.5 ± 3.7	37.18 ± 4.51	44.56	< 0.001	< 0.001	1.000	< 0.001
VR	1.66 ± 0.39	1.43 ± 0.31	1.6 ± 0.39	7.46	0.107	0.081	0.628	0.086
Enghoff index	0.56 ± 0.08	0.48 ± 0.08	0.54 ± 0.08	99.79	< 0.001	< 0.001	0.0158	< 0.001
VD_Bohr_/VT	0.49 ± 0.07	0.41 ± 0.06	0.48 ± 0.07	102.5	< 0.001	< 0.001	0.3304	< 0.001
VT_alv_ /VT	0.64 ± 0.074	0.71 ± 0.076	0.65 ± 0.076	73.21	< 0.001	< 0.001	0.727	< 0.001
VT_alv_/VTeffective	0.51 ± 0.07	0.59 ± 0.07	0.53 ± 0.07	67.11	< 0.001	< 0.001	0.1382	< 0.001
VCO2 (mL·min^−1^)	191 ± 34	227 ± 39	212 ± 44	36.56	< 0.001	< 0.001	< 0.001	0.003
SnIII (L^−1^)	0.072 ± 0.015	0.067 ± 0.014	0.066 ± 0.016	7.16	0.006	< 0.001	< 0.001	0.569

Continuous variables are expressed as the mean and standard deviation (± SD). The F statistic and its corresponding p-value were reported to assess the overall effect of time. A post hoc analysis was performed using pairwise contrasts based on estimated marginal means (EMMeans) to identify specific differences across time points (comparisons T1 vs. T2, T1 vs. T3, and T2 vs. T3). VT = tidal volume; RR = respiratory rate; V̇E = minute ventilation; V̇E = minute ventilation; C_RS_ = respiratory system compliance; PEEP = positive end-expiratory pressure; PaO_2_/FIO_2_ = arterial oxygen tension to inspired oxygen fraction ratio; PaCO_2_ = arterial carbon dioxide tension; V̇Talv = alveolar tidal volume; PACO_2_ = mean alveolar carbon dioxide tension; VR = ventilatory ratio; VD_Bohr_/VT = Bohr dead-space fraction; V̇Talv/VT = alveolar ventilation fraction; V̇Talv/VT effective = effective alveolar ventilation fraction (corrected for alveolar dead space); V̇CO_2_ = carbon dioxide output; SnIII = normalized phase III slope of the capnogram.

**Fig. 1:**
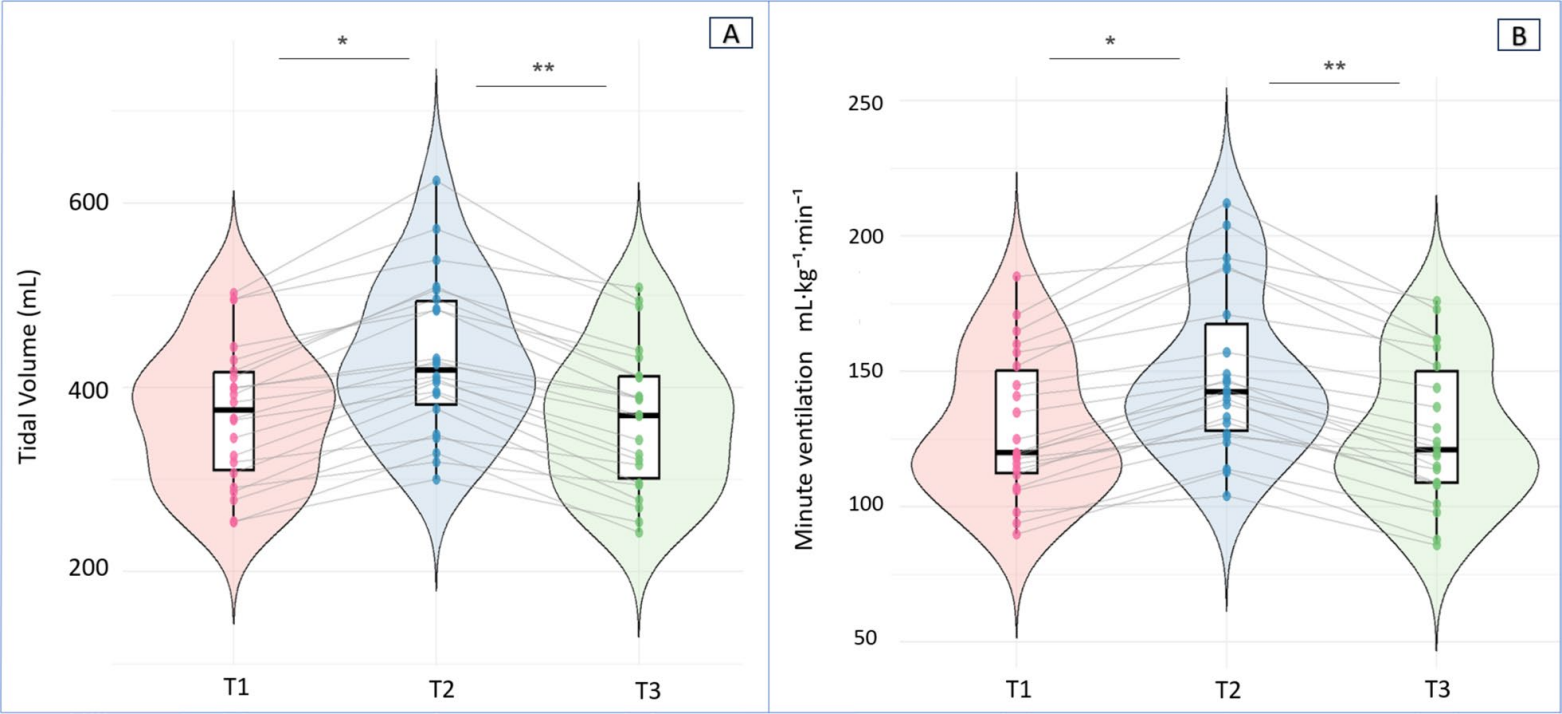
Changes in tidal volume (**A**) and minute ventilation (B) across the three sequential experimental phases. Data are presented as scatter–box–violin plots summarizing all paired observations. The boxes represent the interquartile range (25th–75th percentiles), whiskers denote the 10th–90th percentiles, and the horizontal line indicates the median. Post hoc pairwise comparisons with Bonferroni correction: *p < 0.05 for T2 vs. T1; *p < 0.05 for T3 vs. T2

From Time 1 (baseline) to Time 2 (intervention), VT increased by 62 ± 24 mL (16 ± 6%), and V̇E increased by 1.25 ± 0.37 L·min⁻^1^ (15.5 ± 6.1%). From Time 2 to Time 3 (return to baseline), VT decreased by 68 ± 25 mL (− 15.7 ± 6.2%), and V̇E decreased by 1.38 ± 0.37 L·min⁻^1^ (− 15.7 ± 6.2%), (Fig. 1A, Fig. 1B).

**Fig. 2:**
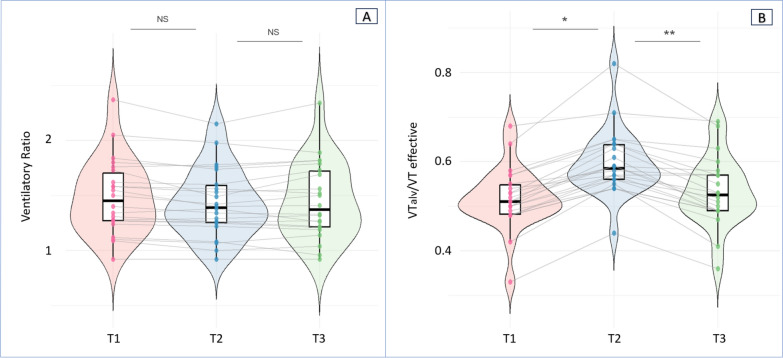
Changes in the ventilatory ratio (**A**) and the effective alveolar ventilation fraction (VTalv/VT effective) (**B**) across the three sequential experimental phases. Data are presented as scatter–box–violin plots summarizing all paired observations. Boxes represent the interquartile range (25th–75th percentiles), whiskers denote the 10th–90th percentiles, and the horizontal line indicates the median. Post hoc pairwise comparisons with Bonferroni correction: *p < 0.05 for T2 vs. T1; *p < 0.05 for T3 vs. T2

○ Ventilatory ratio (VR): from Time 1 to Time 2, Δ =  − 0.23 (95% CI, − 0.44 to − 0.02; *p* = 0.071); from Time 2 to Time 3, Δ =  + 0.17 (95% CI, − 0.04 to + 0.38; *p* = 0.086) (Fig. 2A).
○ Ventilatory efficiency (V̇Talv/VTeffective): from Time 1 to Time 2, Δ =  + 0.080 (95% CI, + 0.039 to + 0.121; *p* < 0.001); from Time 2 to Time 3, Δ =  − 0.060 (95% CI, − 0.101 to − 0.019; *p* < 0.001) (Fig. 2B).
○ Ventilatory inefficiency (VD_Bohr_/VT): from Time 1 to Time 2, Δ =  − 0.080 (95% CI, − 0.119 to − 0.042; *p* < 0.001); from Time 2 to Time 3, Δ =  + 0.070 (95% CI, + 0.032 to + 0.109; *p* < 0.001).

**Fig. 3:**
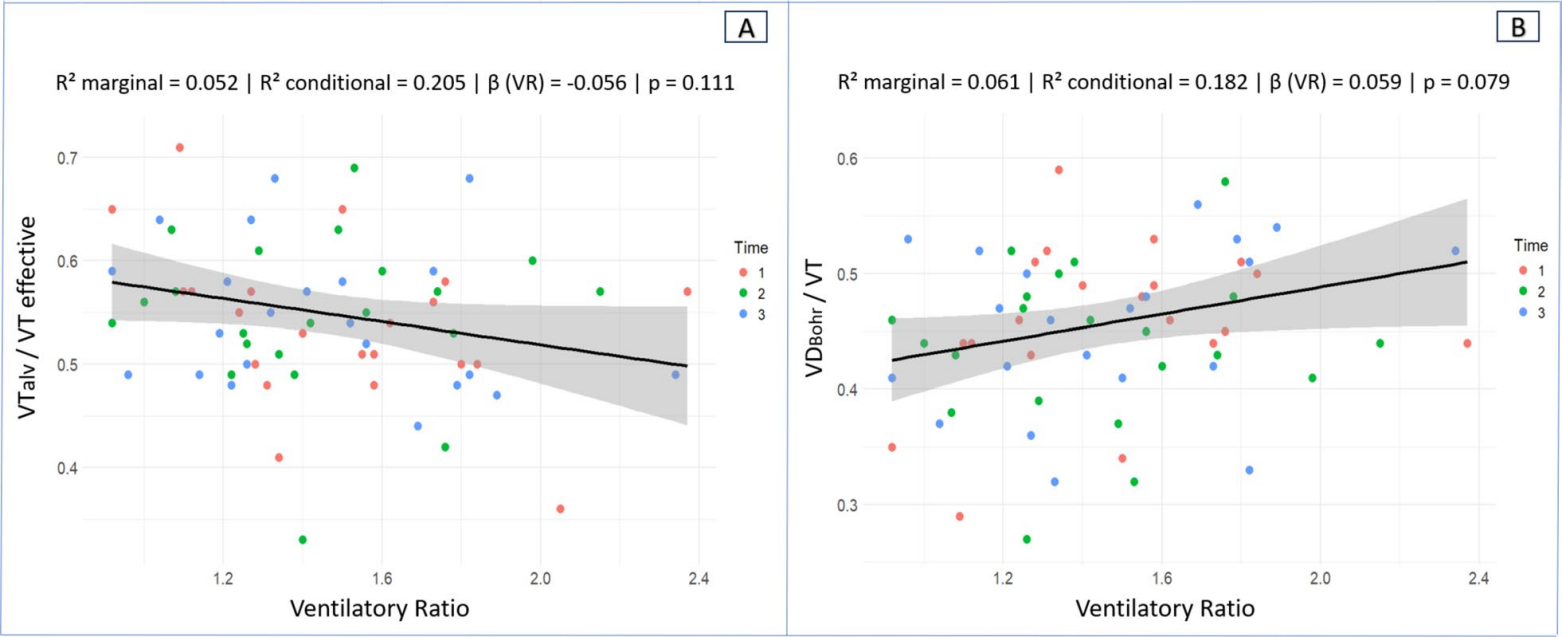
Scatter plot with regression line. **A** Association between Ventilatory Ratio and effective alveolar ventilation fraction (VT_alv_/VT effective). **B** Association between VR and the Bohr dead space fraction (VD_Bohr_/VT). Each dot represents an individual measurement color-coded by experimental time (red = Time 1, green = Time 2, blue = Time 3). The solid black line represents the fitted linear regression model, and the shaded gray area denotes the 95% confidence interval of the regression estimate

**Fig. 4:**
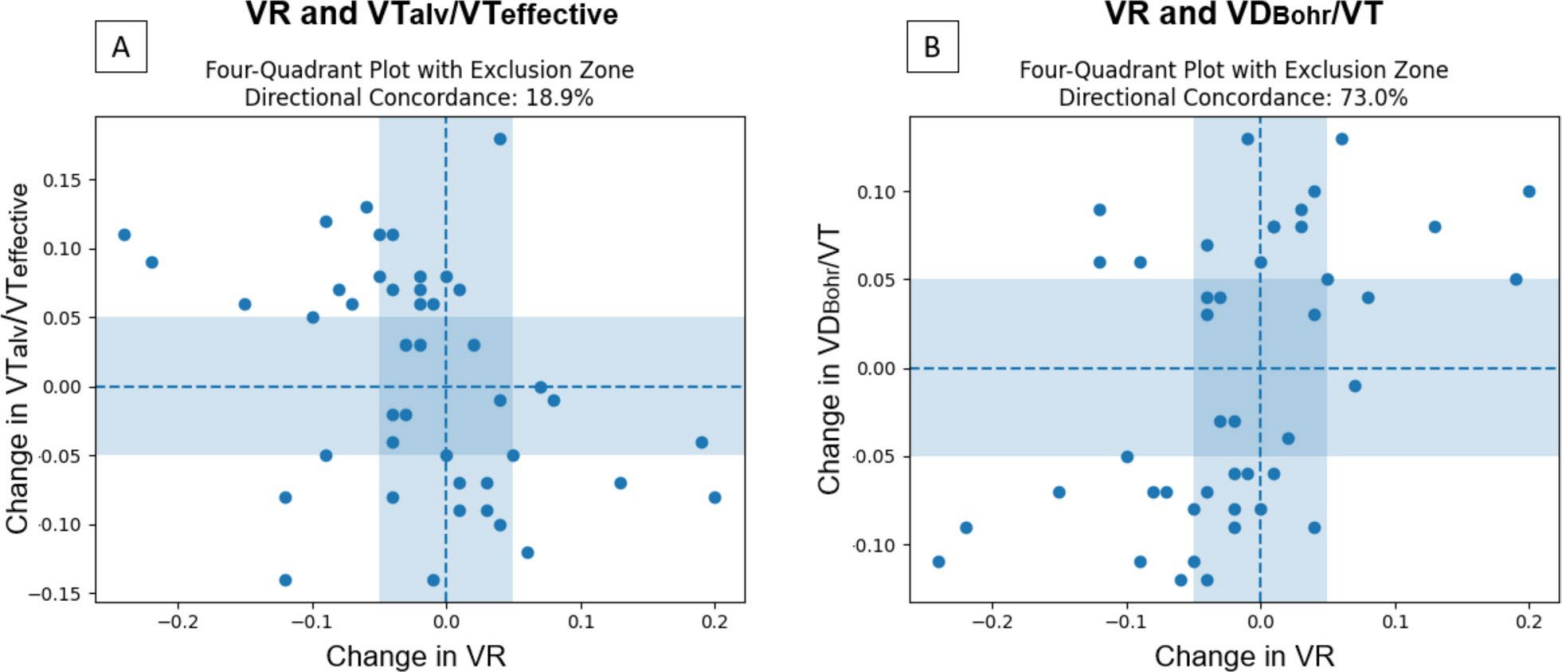
Four-quadrant plots with exclusion zones assessing directional concordance between the ventilatory ratio (VR) and indices of ventilatory efficiency and inefficiency across bidirectional postural transitions (T1–T2 and T2–T3). Each point represents an individual paired change within a patient. Dashed lines indicate zero changes in each variable. The shaded central area corresponds to the predefined exclusion zone (± 0.05 units on both axes), and directional concordance was calculated using only the observations outside this zone. **A** Concordance between changes in VR and V̇Talv/VTeffective. **B** Concordance between the changes in VR and VD_Bohr_/VT

VR was not associated with ventilatory efficiency, as assessed by V̇Talv/VTeffective (β =  − 0.056; marginal R^2^ = 0.052; conditional R^2^ = 0.205; p = 0.111). When V̇Talv/VTeffective decreased by 0.10 units, VR increased by 0.006 units. (Fig. 3A). Likewise, concordance analysis using four-quadrant plots incorporating both T1–T2 and T2–T3 transitions demonstrated poor directional agreement between VR and V̇Talv/VTeffective, with a concordance rate of 18.9%, indicating that changes in VR did not consistently track changes in ventilatory efficiency at the individual level (Fig. 4A).

VR was not associated with ventilatory inefficiency as assessed by VD_Bohr_/VT) (β = 0.059; R^2^ marginal = 0.061; R^2^ conditional = 0.182; p = 0.079). When VD_Bohr_/VT increased by 0.10 units, VR rose by 0.006 units (Fig. 3B). The analysis comparing VR with VD_Bohr_/VT demonstrated a concordance rate of 73%, reflecting moderate directional agreement between the two variables over time (Fig. 4B).

**Fig. 5:**
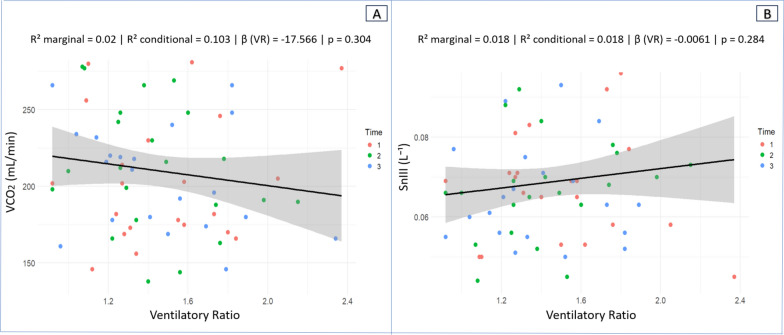
Scatter plot with regression line. **A** Association between Ventilatory Ratio and Minute CO₂ output (V̇CO₂). **B** Association between VR and the Phase III slope (SIII) of the capnogram normalized to the fraction of expired CO₂ (F_E_CO₂) = SnIII. Each dot represents an individual measurement color-coded by experimental time (red = Time 1, green = Time 2, blue = Time 3). The solid black line represents the fitted linear regression model, and the shaded gray area denotes the 95% confidence interval of the regression estimate

VR was not significantly associated with V̇CO₂ (β =  − 17.566; marginal R^2^ = 0.020; conditional R^2^ = 0.103; *p* = 0.304). For reference, an increase of 100 mL·min⁻^1^ in V̇CO₂ corresponds to an estimated ~ 1.76-unit decrease in VR (Fig. 5A). VR was not significantly associated with SnIII (β = 0.0061; marginal R^2^ = 0.018; conditional R^2^ = 0.018; *p* = 0.284). For reference, a 0.01-unit increase in SnIII corresponds to an estimated ~ 0.16-unit increase in VR (Fig. 5B).

VR showed a non-significant positive association with VD_Enghoff_/VT (β = 0.0397, marginal R^2^ = 0.021, conditional R^2^ = 0.037, *p* = 0.256). For reference, a 0.10-unit increase in VD_Enghoff_/VT corresponds to an estimated ~ 0.004-unit increase in VR.

## Discussion

The main findings of this secondary analysis indicate that changes in VT and V̇E were not accompanied by parallel shifts in VR, despite dynamic changes in alveolar ventilation when respiratory mechanics were altered. These observations challenge the physiological validity of VR as a surrogate marker of ventilatory efficiency.

Data used in this analysis comes from a repeated-measures framework, in which posture-induced transitions elicited consistent increases in VT and lung mechanics without modifications in driving pressure, PEEP, or sedation level. This configuration effectively constituted a natural experiment, isolating the impact of body position on alveolar ventilation, independent of ventilator adjustments. In the original study, robust and reversible effects on capnography-based dead-space indices and CO_2_ clearance were demonstrated once patients were in the supine-flat position, and the effects vanished upon returning to the 45° position, validating the internal reproducibility of the physiological response [10].

VR has been proposed as a clinically practical bedside index for assessing ventilatory efficiency [1–3, 13]. Previous investigations that validated VR relied on computing the Enghoff dead space using PaCO_2_ as a surrogate for the PACO_2_ [1–3, 6, 7, 13]. However, in patients with ARDS, PaCO_2_ does not necessarily reflect PACO_2_, because it is influenced by ventilation–perfusion mismatch (e.g., shunting and venous admixture) [7, 13], leading to systematic overestimation of dead space [14–16]. Moreover, VR correlates weakly with minute CO_2_ production (r = 0.07), which is an accurate ventilatory efficiency marker [2]. In contrast to these studies, we directly measured PACO_2_, which provided a more accurate assessment of ventilatory efficiency [1, 16]. Using this approach, we observed only weak associations between VR and V̇Talv/VTeffective and between VR and VD_Bohr_/VT. In parallel, concordance analyses incorporating bidirectional transitions (Time 1–Time 2 and Time 2–Time 3) were performed to provide additional physiological insights beyond the conventional association metrics. VR and V̇Talv/VTeffective are expected to change in opposite directions during respiratory impairment, which is consistent with the poor directional concordance observed in the four-quadrant analysis. In contrast, VR showed moderate directional concordance with indices of ventilatory inefficiency, such as VD_Bohr_/VT, which are physiologically expected to vary in the same direction as VR. However, concordance reflects direction rather than strength of association, and despite this moderate directional agreement, the magnitude of change in VD_Bohr_/VT associated with VR was small and of limited clinical relevance.

The mathematical structure of VR also limits its physiological accuracy. Its denominator assumes a normalized V̇E of 100 mL·kg⁻^1^·min⁻^1^, a constant derived from anesthetized non-ARDS populations [1, 9]. In contrast, critically ill patients typically exhibit significantly higher ventilatory demands (≈ 150 mL·kg⁻^1^·min⁻^1^) [9]. Therefore, the consistent association between VR and adverse clinical outcomes reported in multiple studies [17–20] could be explained by the action of other factors implicit in the VR equation, such as drive and ventilatory load [21], metabolic production of CO_2_ [22, 23], and impairment of cardiac output [14, 22].

Most previous studies evaluating VR as a marker of ventilatory efficiency have used cross-sectional designs [2, 3, 13]. In contrast, we employed a repeated-measures design to accurately characterize the variations in alveolar ventilation during passive mechanical ventilation, using each patient as their own control [10]. Our findings show a dissociation between changes in VR and ventilatory efficiency after changes in position. These results are consistent with those of a previous study that examined the effects of trunk inclination in obese and nonobese patients with ARDS using a similar study protocol design [24]. Although postural adjustments induced significant changes in respiratory mechanics, PaCO_2_, and Bohr dead space, particularly in patients with a body mass index ≥ 30 kg/m^2^, these effects were not accompanied by variations in the VR under either of the clinical conditions studied [24].

In studies that used volumetric capnography to compute dead space and compared it with VR, the number of breaths averaged per measurement was rarely specified, thereby limiting the interpretation of these findings [2, 3, 8, 13]. This omission is particularly relevant because, even under controlled mechanical ventilation, breath-to-breath variability in respiratory variables can be considerable [25]. To minimize this variability, our study averaged 20 consecutive breaths for each measurement, ensuring stable, representative estimates of gas exchange and quality of data collection, thereby strengthening the validity of our results [6, 7, 10]. Thus, in general terms, our study provides a rigorous physiological framework for assessing whether VR truly reflects changes in ventilatory efficiency. This approach establishes a methodological foundation necessary to clarify the physiological significance and limitations of VR in patients on mechanical ventilation.

### Final comments

This study demonstrated that short-term modifications in respiratory mechanics and the resulting changes in alveolar ventilation do not lead to significant alterations in the VR. Therefore, fluctuations in VR over days and their association with poor clinical outcomes in clinical practice [2, 4] may stem from several uncharacterized factors. For instance, it remains to be determined how metabolic CO_2_ production (V̇CO_2_) and changes in cardiac output influence or modify VR and shape its relationship with mortality. Accordingly, VR is likely best interpreted as an integrated compass of ventilatory–metabolic load and perfusion rather than a “pure” measure of alveolar or ventilatory efficiency, as previously assumed. Finally, our findings do not question the prognostic value of VR but rather clarify its physiological interpretation under controlled conditions.

### Limitations

This study had several limitations. First, the small sample size and single-center design may limit the generalizability and reproducibility of the findings across different patient populations and clinical settings. Although the results are consistent with our a priori hypothesis that VR is not meaningfully associated with V̇Talv/VTeffective, post hoc power analysis suggests that detecting the effects of the observed magnitude with 80% statistical power would require a larger cohort (n = 80–90). However, the present study provides physiologically grounded effect-size estimates derived from a controlled, repeated-measures design. These estimates provide a robust basis for informing a priori sample size calculations in future prospective studies aimed at rigorously evaluating the physiological relationship between VR and ventilatory efficiency.

Second, this study represents a secondary analysis of a study not originally designed to validate VR as a physiological marker, which may introduce cohort-related selection bias and limit causal inference [26]. Accordingly, while the repeated-measures quasi-experimental design strengthens internal validity, the present findings should be interpreted within a physiological framework and regarded as hypothesis-generating, warranting confirmation in larger prospective multicenter studies.

Third, the effects of changes in VT and V̇E on VR were assessed over a brief interval (60 min per phase); thus, the findings reflect a short observation period. The changes in VT and V̇E were the result of physiological responses to trunk inclination and varied across patients, influenced by intrinsic factors such as the etiology of acute respiratory failure and an increased body mass index (BMI ≥ 30 kg/m^2^) [24]. Although the hemodynamic variables were held constant, we cannot exclude the possibility that inclination-induced changes in VT were accompanied by global and/or regional changes in pulmonary blood flow and, consequently, in expiratory CO_2_ clearance. Although the multiple inert gas elimination technique (MIGET) remains the reference method for PACO_2_ estimation, volumetric capnography-derived PACO_2_ values closely correlate with those obtained from reference methods.

## Conclusions

In this cohort of ARDS patients, we observed that VR was not associated with V̇Talv/VT following modifications to respiratory mechanics. These findings suggest that VR may be an imprecise proxy for ventilatory efficiency, cautioning against its use as a surrogate for ventilatory efficiency when ventilatory conditions are modified. Further mechanistic studies are needed to establish the respective contributions of alveolar ventilation, metabolism, and perfusion (cardiac output/VQ) to VR and to determine whether VR should be used to inform bedside decision-making. These results should be confined to physiological interpretations and considered hypothesis-generating.

## Data Availability

The data sets used and analysed during the current study are available from the corresponding author upon reasonable request.

## Abbreviations

ARDS: Acute respiratory distress syndrome
BMI: Body mass index
CI: Confidence interval
C_RS_: Respiratory system compliance
FECO_2_: Fraction of expired carbon dioxide
ICU: Intensive care unit
PACO_2_: Alveolar carbon dioxide pressure
PaCO_2_: Arterial carbon dioxide pressure
PBW: Predicted body weight
PECO_2_: Mean expired carbon dioxide pressure
PEEP: Positive end-expiratory pressure
RR: Respiratory rate
SD: Standard deviation
SIII: Phase III slope of the capnogram
V/Q: Ventilation–perfusion ratio
V̇A: Alveolar minute ventilation
V̇CO_2_: Minute carbon dioxide output
V̇E: Minute ventilation
VD_Bohr_/VT: Bohr dead-space fraction
VD_Enghoff_/VT: Enghoff dead-space fraction
VDalv: Alveolar dead space
VDaw: Airway dead space
VR: Ventilatory ratio
VT: Tidal volume
VTCO_2_,_br_: Carbon dioxide eliminated per breath
V̇Talv/VT: Alveolar ventilation fraction
V̇Talv/VT: Effective effective alveolar ventilation fraction
